# Histotripsy: A novel non-invasive ultrasound technology for precision thrombolysis in acute ischemic stroke management

**DOI:** 10.1177/15910199251347838

**Published:** 2025-06-10

**Authors:** Ameer E Hassan, Thomas Dreyer, Yousef Khalili

**Affiliations:** 1 Department of Neurology, Valley Baptist Medical Center/University of Texas Rio Grande Valley, Harlingen, TX, USA; 2 Quantanosis.AI, LLC. Austin, TX, USA; 3 Weber Ultrasonics AG, Karlsbad, Germany

**Keywords:** Histotripsy, stroke, thrombolysis, ultrasound, mechanical thrombectomy

## Abstract

**Background:**

Stroke remains a major cause of morbidity and mortality worldwide. Traditional treatments for ischemic stroke such as mechanical thrombectomy and pharmacological thrombolysis are often less effective or inaccessible for certain patient populations, particularly those with delayed hospital arrival, contraindications to thrombolytics, or in regions with limited access to endovascular care. This study investigates the feasibility of using histotripsy, a non-invasive ultrasound technology, to precisely liquefy thrombi in an artificial experimental setup.

**Methods:**

Clot analogs were introduced into sample tubes, and focused ultrasound was applied using optimized parameters, including pulses per burst, repetition rates, and output amplitudes. These parameters were systematically adjusted to determine the most effective settings for clot ablation, with a focus on maximizing clot liquefaction while minimizing residual fragments.

**Results:**

Histotripsy achieved complete clot liquefaction within 60–120 s at a repetition rate of 100  Hz, with 1000 pulses per burst and an output amplitude of 100%. The procedure effectively disintegrated soft (red blood cell-rich) clots, leaving minimal residual fragments (<250 microns). However, dense fibrin-rich clots and anatomical barriers like bone presented challenges, suggesting the need for further optimization.

**Conclusions:**

This proof-of-concept study demonstrates the potential of histotripsy as a non-invasive, rapid, and targeted approach for clot removal in stroke management.

## Introduction

Stroke presents a significant global healthcare burden, with ischemic stroke accounting for approximately 80% of all strokes.^1^ In the United States approximately 795,000 strokes occur annually, most of which are ischemic.^2^ Just 1.6%–6.9% of ischemic stroke patients are eligible for intravenous tissue plasminogen activator (tPA) due to the narrow therapeutic window.^3,4^ Only 2.6% of individuals worldwide have access to mechanical thrombectomy,^5^ and many hospitals in rural areas lack the resources or trained personnel to perform thrombectomies, leading to inequities in care.^6^ Furthermore, some patients are ineligible for mechanical thrombectomy due to time since stroke occurrence, presence of comorbidities, coagulopathy, large infarct, bleeding risk, advanced age, or contraindications to anesthesia.

Histotripsy, a novel non-invasive ultrasound technique, offers a promising alternative for clot disintegration and may be more widely deployable than mechanical thrombectomy. Studies have shown that ultrasound can enhance thrombolysis by improving tPA-mediated clot breakdown.^7–9^ Use of ultrasound during thrombolytic treatment can enhance clot lysis via cavitation, which perturbs the thrombus and exposes additional fibrin binding sites for tPA.^10–12^ Furthermore, combining ultrasound with tPA therapy has been shown to enhance recanalization in patients with acute ischemic stroke.^8^ The potential of ultrasound as a non-invasive therapy is further supported by studies demonstrating its efficacy in various thrombolytic scenarios, including high-intensity focused ultrasound (HIFU) applications for clot dissolution in both in vitro and in vivo settings.^13,14^ The efficacy of ultrasound-assisted thrombolysis in treating ischemic stroke is an area of active research.^15–18^

This study evaluated the feasibility of using histotripsy to liquefy thrombi in an artificial experimental setup. By optimizing ultrasound parameters including pulses per burst, repetition rates, and output amplitudes, this study identified the most effective settings for clot ablation and assess the potential of histotripsy as a targeted, rapid, and non-invasive treatment for stroke.

## Methods

Data supporting the findings of this study are available from the corresponding author upon reasonable request. The corresponding author had full access to all the data in the study and takes responsibility for its integrity and analysis.

### Histotripsy device

An experimental HIFU system was designed for non-invasive tissue ablation (Figure S1). The system features a single focusing transducer of spherical cap geometry with a geometrical focal length (radius) of 75 mm and active diameter of 100 mm (aperture), capable of delivering precise, high-energy ultrasound pulses at a frequency of 1.5 MHz. The transducer was mounted in a water tank, allowing for effective ultrasound wave coupling to the clot sample tubes, which were positioned using a 3-axis precision positioning stage at the transducer's focal point to maximize energy delivery (Figure 1). The system allows adjustable parameters, including pulses per burst, repetition rate, and output amplitude. It also supports the use of gel pads affixed to the transducer to simulate an acoustic coupling mechanism, as would be required in a clinical setting to enhance energy transmission when anatomical barriers such as bone are present. In some experiments, two custom shaped gel pads (AQUAFLEX^®^, Parker Labs, Fairfield, NJ) were mounted in front of the transducer, where the first fills the complete spherical cap, and the second is cylindrical (90 mm diameter, 10 mm thickness) is placed on top of the first.

**Figure 1.**
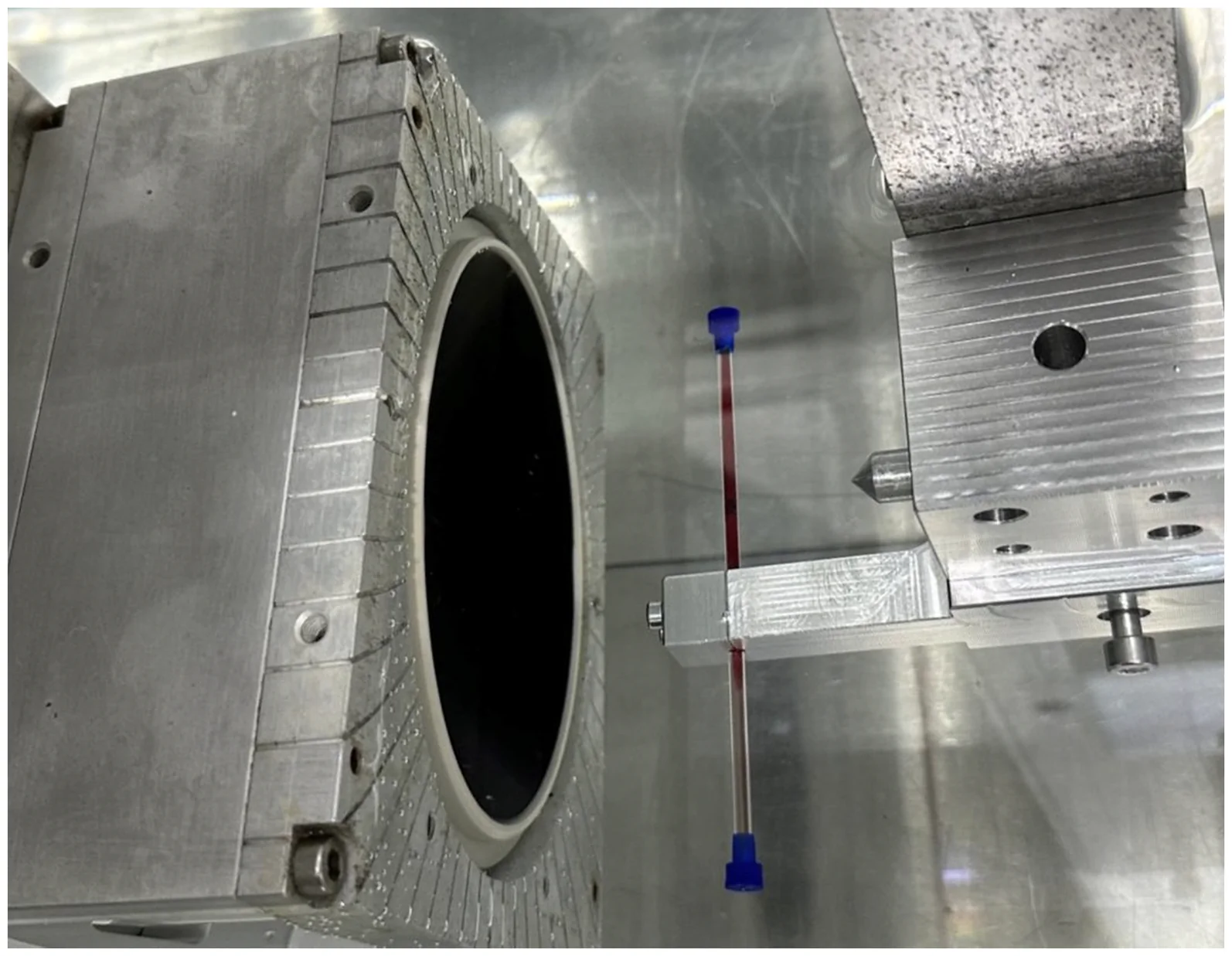
Test setup with transducer (left) and glass tube.

Sample tubes (Hilgenberg, Germany) are made of glass with 3 mm outer diameter, 2.4 mm inner diameter, and 100 mm length. The impact of the tube on the ultrasonic field was minimized due to the small wall thickness (0.3 mm) and the fact that the tubes were liquid filled, thus avoiding scattering through acoustic impedance mismatch.

Tests were conducted with sample tubes placed at angles of 10°, 20°, and 30° relative to the transducer to assess whether the angle affects treatment efficacy. Additionally, a dual transducer setup was tested to explore whether using two ultrasound transducers with different focal settings could improve clot dissolution

### Preparation of clot samples

Clot analogs were sourced from Cerenovus, Johnson & Johnson using a standard formulation^19^ to mimic thrombi encountered in ischemic stroke. Clots were sliced into uniform pieces measuring 5–6 mm in length and approximately 2 mm in diameter. Pieces were placed into glass tubes with an inner diameter designed to simulate the middle cerebral artery. Tubes were filled with saline using a syringe to ensure clots remained fully submerged and hydrated. After preparation, tubes were sealed at both ends to prevent leakage and stored at 4°C. The storage life of clots is 14 days at 4°C. Experiments with glass tubes were conducted before expiry. Clots used for experiments with the glass model were used within a maximum of 10 days beyond the expiration date.

### Histotripsy procedure

Clot samples were positioned within the transducer's focal region inside the water tank and held at a fixed position during treatment (Figure 1). The transducer generated short bursts of high-energy ultrasound to disrupt the clot material through cavitation—a process where microbubbles are created and then collapse, mechanically breaking apart the clot. System parameters were optimized across multiple treatment tests to determine the ideal settings for clot disruption, focusing on pulse number, repetition rate, and output amplitude. Clot fragmentation (Figure 2) was monitored visually during experiments. High-resolution pictures of the samples were taken pre-and post-treatment.

**Figure 2.**
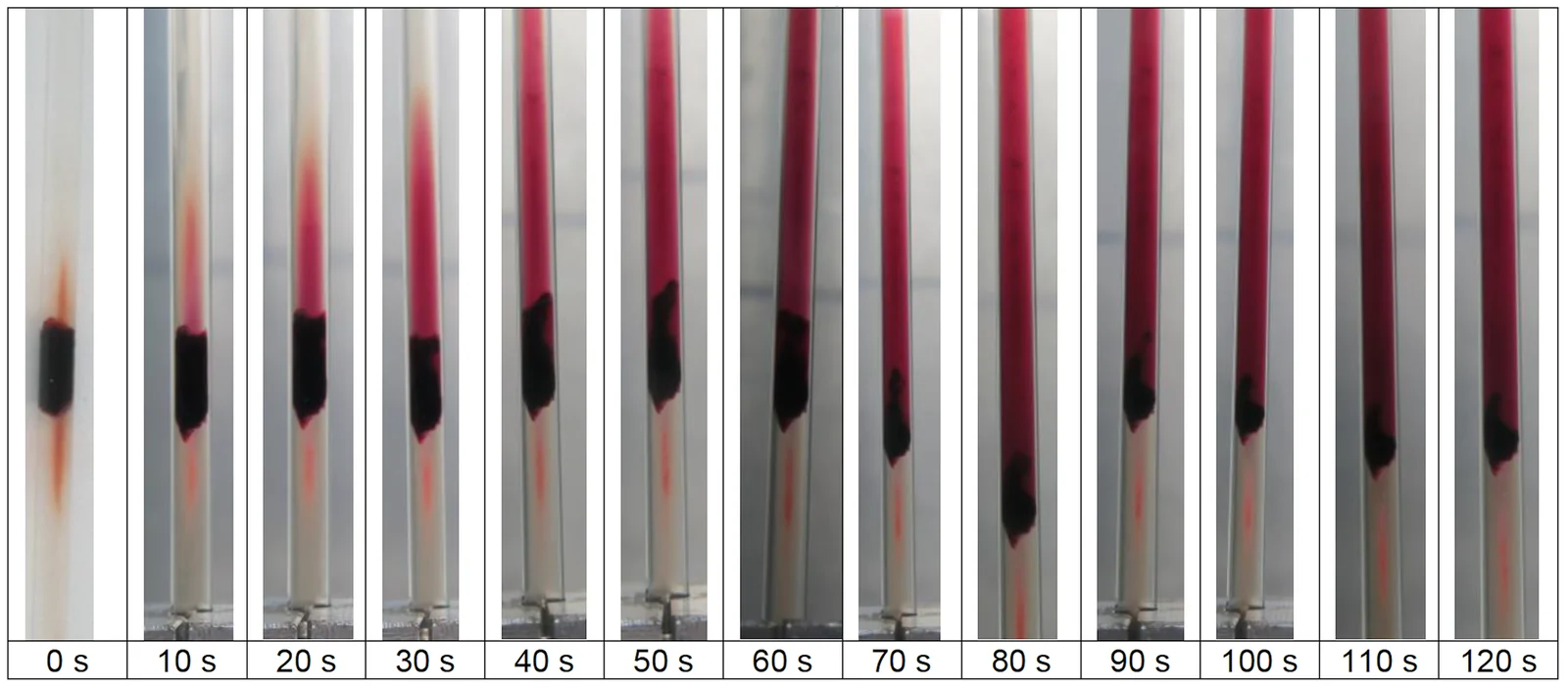
Time-lapse of clot dissolution.

In some experiments, an anatomically correct glass model simulating more complex human vasculature was used (Figure S2). This setup aimed to simulate real-world anatomical environments and assess the feasibility of focused ultrasound treatments in more complex conditions. The glass model replicated the dimensions of key vessels like the middle cerebral artery. Its transparent walls allowed real-time observation of clot positioning and dissolution during histotripsy, helping to assess ultrasound transmission and effectiveness in a realistic anatomical setting.

### Quantification of clot destruction

A Canon Powershot G9X (20.2 MP, 3x optical zoom, 4x digital zoom) camera was used to capture images of clot fragments pre- and post-treatment and image processing software (developed in-house) was used to assess the number and size of the particles. The methodology is based on the assumption that hemoglobin is concentrated to the clot before destruction and diluted in the tube after. Diluted hemoglobin appears as lighter red pixels compared to the dark red pixels in the clot. Any unresolved darker remains were analyzed. This process allowed for precise measurement of the clot fragments’ dimensions before and after treatment. By comparing the particle size and volume, the relative size reduction (RSR) was calculated using clot pixel counts (CPC), quantifying the extent of clot dissolution achieved in each trial:

$$RSR=\left(1-\begin{array}{c} CPC_{\;post}\\ CPC_{\;pre} \end{array}\right)\times 100\text{\%}\;$$

An example of the applied image processing method is shown in Figure S3. Quantification of clot destruction provided insights into the effectiveness of each ultrasound parameter in disrupting the clots.

### Ultrasound parameters and optimization

Measurements of the ultrasonic output were performed following the definitions and methods described in IEC Technical Specification 62556:2014.^20^ Ultrasonic signals were measured in a water tank shown in Figure 2 in deionized and partially degassed water using a fiber-optical probe hydrophone (FOPH 2000, RP Acoustics, Leutenbach, Germany). The hydrophone was calibrated according to the procedure prescribed by the manufacturer and calibration was checked regularly before, during and after measurements. Because of its operational principle, the fiber optic probe hydrophone has a linear frequency response up to 100 MHz with an overall measurement uncertainty of ±10% for pressure amplitude values.^21^ Spatial distribution of the pressure amplitudes was also recorded by measuring along the center axis of the transducer to identify the acoustical focus position. The lateral amplitude distribution was then recorded for the focal point to calculate the focal dimensions, which are defined as −6 dB decrease of the pressure pulse squared intensity integral.^22^

The output amplitude represents the amplitude of the ultrasound waves emitted by the transducer. In this study, the output amplitude was varied between 75% and 300% to assess its impact on clot dissolution, where the value of 100% represents the maximum amplitude for applications in water as a reference value. Higher values provide additional headroom for applications where additional absorption or reflection of the emitted wave is inserted in the propagation path. Lower amplitudes, such as 75%, were tested to explore the minimum energy required for clot ablation while minimizing potential damage to surrounding tissues. The output amplitude was adjusted during early tests to identify the threshold level required for effective clot disruption. Multiple samples were tested at increasing amplitudes, and the level at which complete clot dissolution was consistently achieved was recorded. The relationship between output amplitude and clot fragmentation was assessed visually and quantified using the RSR metric.

Repetition rate refers to how frequently the ultrasound pulses are emitted, measured in Hertz (Hz). In this study, repetition rates ranging from 20 to 100 Hz were tested to determine the rate that provided the most efficient clot disruption. Repetition rates were systematically adjusted during a series of treatment tests, starting at 20 Hz and increasing incrementally to 100 Hz. Clot dissolution was monitored in real-time, and the effect of different repetition rates on the speed and completeness of clot ablation was compared. The optimal repetition rate was determined by identifying the rate that provided the fastest and most complete clot fragmentation without causing significant scattering of clot material.

Pulses per burst is the number of 1.5 MHz cycles delivered in a single burst. This parameter was varied between 10 and 1000 pulses per burst to assess how the number of pulses in each burst impacted clot fragmentation. The number of pulses per burst was adjusted in several treatment tests, beginning with lower values and gradually increasing to higher values. Clot samples were evaluated for the degree of disruption achieved at each pulses per burst level, with the RSR metric used to quantify the clot size reduction after each trial. The ideal pulses per burst were identified by balancing effective clot disruption with the total number of pulses to avoid excess energy delivery.

The number of bursts represents how many times a series of pulses was emitted during each histotripsy session. This parameter varied between 3000 and 9000 bursts across trials to evaluate the total exposure time needed to achieve full clot dissolution. The number of bursts was varied over a range of treatment tests to determine the minimum number required to achieve complete clot dissolution. Clot samples were exposed to increasing numbers of bursts, and clot ablation was assessed after each session. The optimal number of bursts was determined by identifying the point at which increases in bursts did not significantly improve clot destruction, ensuring efficient energy use without unnecessary exposure.

## Results

### Ultrasonic parameters

Measurements of ultrasound signal were performed at pulse parameters of 5 to 10 pulses per burst at a repetition rate of 1 Hz at different output amplitudes. These parameters were chosen to minimize cavitation effects at high amplitude levels. However, even though the parameters were chosen carefully, it was not possible to record reliable focal pressure signals above an output level of 100%, because of the increasing occurrence of cavitation close to the sensor with increasing output levels. The pressure waveform is shown in Figure 3 for an output amplitude of 75%, whereas pulse amplitudes and shape are comparable to similar applications.^22^ Figure 4 shows peak positive and negative amplitude values for an output setting range from 10 to 100% with maximum peak values of 158 and −23 Mpa, respectively, which are also in the range or even higher than those reported in literature for these applications.^22^ Peak positive amplitudes do not increase significantly at amplitude settings above 80%, which is caused by nonlinear wave propagation where shock fronts have fully developed and pressure amplitudes at higher driving signal amplitudes would only increase to small amounts.^23^ This leads to and supports the assumption that no significant increase in peak focal pressure amplitudes is expected for propagation in water at output amplitude settings above 100%. Focal dimensions at 100% output amplitude result in a lateral diameter of 2.3 ± 0.1 mm and an axial extension of 4.9 ± 0.1 mm. The system's precise focus minimized scatter of ultrasound energy outside the focal zone, ensuring targeted clot disruption.

**Figure 3.**
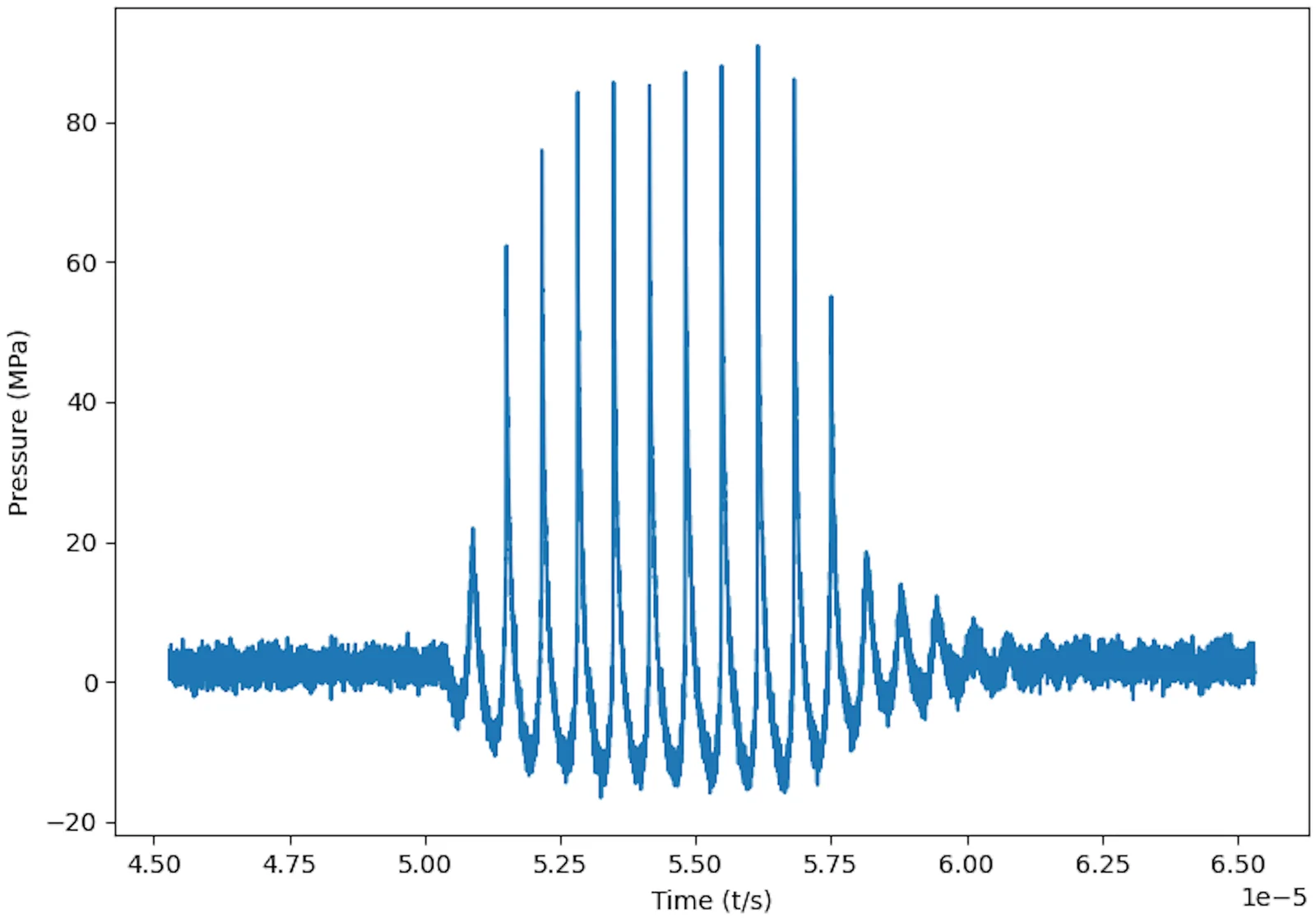
Acoustic pressure waveform in focus at 75% amplitude setting and 10 cycles per pulse.

**Figure 4.**
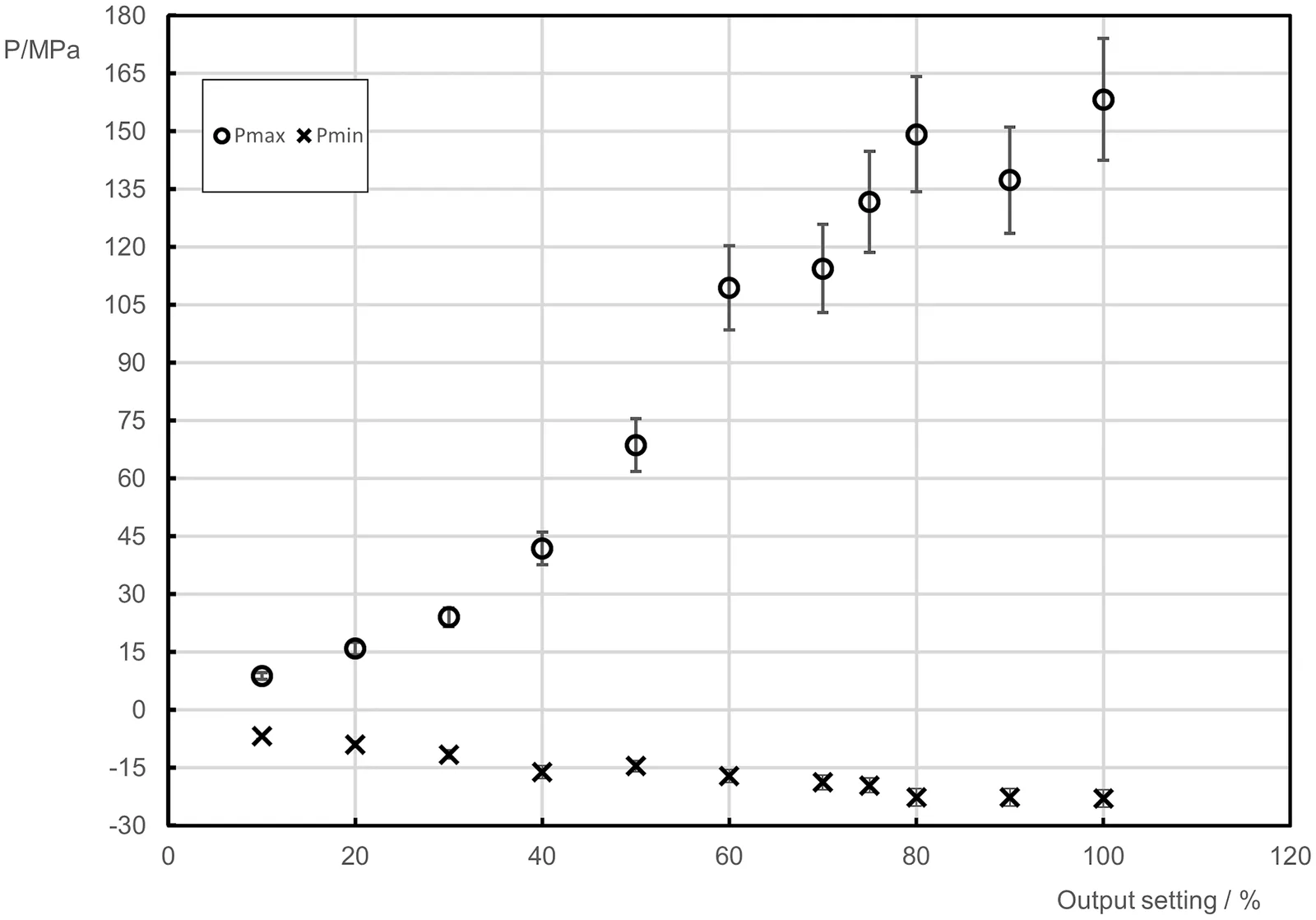
Peak positive and peak negative pressure amplitudes in acoustic focus.

### Pretesting

Early attempts at clot dissolution were unsuccessful, revealing that the tube was entirely filled with clot material. As a result, the cavitation at the focal point generated minimal movement of the clot material, preventing more clot from reaching the focal zone. This led to the clot not being fully destroyed. Another issue occurred when the remaining clot blocked one saline compartment in the tube, preventing the diffusion of clot material in that area. The key takeaway from these early tests was to prepare clots that partially fill the tube, allowing fluid microstreaming caused by cavitation (Figure 1).^24^

### Repetition rate optimization

Initial tests explored a range of repetition rate values from 20 to 100 Hz (Table 1), with at least 3 samples tested per setting. Higher repetition rates yielded faster pulse rates and increased clot interaction, while lower repetition rates allowed for slower but more controlled disruption. We found that lower repetition rate settings (e.g., 20 Hz) were ineffective, with only partial clot destruction. Clots were consistently destroyed at repetition rate = 50 and 100 Hz, with complete dissolution observed at repetition rate = 100 Hz in several tests.

**Table 1.** Summary of results from the repetition rate optimization tests.

Repetition rate (Hz)	Pulses per burst	Output amplitude (%)	Number of bursts	Treatment time (s)	Clot destruction (%)	Observation
20	100	100	2400	120	Partial	Low efficiency, partial clot destruction
50	100	100	6000	120	Complete	Effective, clots fully destroyed
75	100	100	9000	120	Complete	Effective, clots fully destroyed
100	100	100	6000	120	Complete	Most effective, faster complete destruction

In follow-up tests, the treatment time was reduced from 120 to 60 s to investigate the impact of shorter bursts. At repetition rate = 100 Hz, clots were fully destroyed even with a 60-s treatment time, confirming the efficiency of higher repetition rates. At repetition rate = 50 Hz, clots were only partially destroyed during the reduced treatment time, suggesting that repetition rate = 100 Hz performs better when time is limited.

Higher amplitudes increased the intensity of ultrasound pulses, which enhanced the cavitation effect, leading to more efficient clot disruption. Lower output amplitude tests of 75% were conducted to challenge repetition rate values of 75 and 100 Hz. Figure 3 shows an example of the pressure waveform in the acoustical focus of the transducer at an amplitude setting of 75%. These tests revealed that repetition rate = 100 Hz was still more effective than repetition rate = 75 Hz, with all three clot samples being completely destroyed at the higher repetition rate. In contrast, remnants of clots were still visible at repetition rate = 75 Hz under lower output amplitudes. This indicates that repetition rate = 100 Hz is more resilient to reductions in output amplitude, maintaining high effectiveness.

The number of pulses per burst was tested from 10 to 1000. Higher numbers of pulses per burst increased the total energy delivered to the clot, enhancing the cavitation effect and leading to more complete clot ablation. At pulses per burst levels below 200, there was little effect on the clots, with partial or no destruction. At pulses per burst values of 500 or higher, better results were achieved. The study concluded that for effective clot destruction, pulses per burst should be set to 500 or above, with repetition rate values at 75 Hz and 100 Hz showing the most consistent results.

With an output amplitude of 100%, tests focused on combining different pulses per burst and repetition rate settings. Results revealed that the combination of pulses per burst = 500 and repetition rate = 75 Hz produced the best results. This outcome was likely influenced by the small sample size, as inconsistent RSR values were observed across different runs. Despite variations in RSR values, pulses per burst = 500 and repetition rate = 75 Hz were identified as the most promising parameters for further testing at 100% output amplitude.

Initially, no significant impact on clot dissolution was observed with gel pads (Table 2). However, increasing the output amplitude to 300% resulted in instantaneous clot dissolution, verifying that the focus point of the ultrasound remained unchanged despite the gel pad damping. Subsequent tests with an output amplitude of 150% were mostly successful, although degradation of the gel pads over time led to inconsistent results. Gel pads were found to release bubbles and milky clouds when left in distilled water overnight, which affected ultrasound effectiveness.

**Table 2.** Summary of results of tests conducted using gel pads during ultrasound clot dissolution under four test conditions.

Test condition	Output amplitude (%)	Clot destruction	Observation
Initial test	100	No significant impact	Initial tests with gel pads showed no significant effect on clot dissolution.
Increased output amplitude	300	Complete and instantaneous	Instantaneous clot dissolution, confirming focus point effectiveness.
Degradation over time	100	Inconsistent results	Gel pads degraded over time, forming bubbles and clouds, leading to reduced ultrasound effectiveness.
Fresh gel pads test	170	100% destruction, consistent	Freshly mounted gel pads achieved consistent clot destruction in 29 consecutive samples.

To combat gel pad degradation, tests were repeated immediately after mounting new gel pads. In these tests, with output amplitude set to 170%, a 100%, clot dissolution within 30 s was achieved for 29 consecutive samples, suggesting that treatment immediately after mounting fresh gel pads is crucial for optimal outcomes.

Results using an anatomically correct glass model were inconsistent (Table 3). While some cases showed minor clot destruction, most clots either failed to liquefy or were pushed out of focus due to the geometry of the glass model. The glass model's thick walls and complex geometry likely contributed to the lack of efficacy, limiting the transducer's ability to maintain consistent energy focus on the clot. Ineffectiveness in some tests may also have been caused by the age of the mixed clots.

**Table 3.** Summary of results from tests conducted using an anatomically correct glass model for ultrasound clot dissolution, under four test conditions.

Test condition	Observation	Clot destruction
Initial test	Limited success in clot dissolution within the anatomical model.	Minimal
Clot positioning challenges	Difficulties maintaining the clot in the focal point during treatment.	Minimal
Thick wall impact	The thick glass walls hindered ultrasound transmission, reducing effectiveness.	None
Focus area misalignment	Clot misalignment caused the treatment to be less effective.	None

Using a repetition rate of 100 Hz, pulses per burst of 1000, and an output amplitude of 100%, complete clot dissolution was achieved in most test samples within 60–120 s. In clots with complete dissolution, all fragments were smaller than 250 µM. This high success rate demonstrates the reliability of the chosen parameters for clot destruction. Minimal variability was observed across repeated treatment tests, confirming that the method can be reliably reproduced under controlled conditions. When complete clot destruction did not occur, variability was generally attributed to minor setup deviations, such as slight misalignment of the sample or timing fluctuations.

## Discussion

This study demonstrated that focused ultrasound can non-invasively liquefy clots in an artificial set-up with a high degree of precision. This finding is supported by other studies exploring ultrasound applications in thrombolysis,^25,26^ indicating that focused ultrasound can enhance clot dissolution while minimizing damage to surrounding tissues.

### Optimizing parameters for clot dissolution

This study optimized ultrasound parameters for efficient clot destruction, finding that higher repetition rates and more pulses per burst significantly improved clot dissolution. A repetition rate of 100 Hz and 1000 pulses per burst produced the most consistent and complete dissolution. Similarly, previous studies investigating varied pulse rate frequencies ranging from 1 to 1000 Hz in porcine blood clots administered for 30 s found that thrombolysis efficiency increased between 1, 10, and 100, but then significantly dropped in efficiency at 1000 Hz,^27^ emphasizing the importance of precise ultrasound parameter control.^28^ In a study of craniectomized pigs, histotripsy delivered through a transducer with a center frequency of 1.5 MHz with peak rarefactional pressures of 45 mPa and histotripsy pulses at a fixed rate of 10 Hz generated well-defined lesions in the cortex.^29^ In a porcine model of intracerebral hemorrhage, histotripsy treatments delivered using ultrasound pulses with peak-negative pressures of 30–40 mPA at pulse-repetition frequencies of 40–60 Hz liquefied the center of clots without damaging surrounding brain tissue.^30^ In this study, a 100 Hz repetition rate and 1000 pulses per burst completely destroyed clots within 60–120 s. These parameters were particularly effective when combined with an output amplitude of 100%. While reducing the output amplitude to 75% still resulted in some clot destruction, dissolution was less consistent, particularly at lower repetition rates.

### Impact of clot characteristics and medium

A challenge highlighted in the study was the impact of the physical setup on the success of clot dissolution. Clot shape and size, clot age, and clot density are vital to histotripsy outcomes. Soft, red blood cell-rich clots were easily disrupted, while dense, fibrin-rich clots displayed resistance. Clots stored beyond 10 days displayed diminished responses due to hardening. Inconsistent results were observed in glass model experiments, likely due to clot rigidity over time. Thus, it appears that older clots become denser and more resistant to cavitation-induced fragmentation and require higher amplitudes or alternative strategies.

Ultrasound attenuation occurs through dense tissues or the presence of boundaries (e.g., bone plate or anatomical models), impacting treatment outcomes. Others have found that clot geometry and surrounding anatomy impact ultrasound thrombolysis effectiveness.^26^ When the sample tube was angled (e.g., 10°, 20°, 30°), ultrasound still liquefied clots, suggesting that changes in geometry do not impact on clot removal. Introducing a bone plate blocked clot destruction, even at high amplitudes (400–450%), likely due to ultrasound absorption or reflection, suggesting that treating clots near bone or dense tissue may require alternative strategies like multiple transducers or enhanced energy delivery.

### Clinical implications and limitations

Mechanical thrombectomy carries risks such as vessel damage and hemorrhage, and it is typically limited to large vessel occlusions. Thrombolytic drugs are associated with a risk of bleeding and have a narrow therapeutic window, limiting their use in patients with a history of hemorrhagic stroke, bleeding disorders, or recent surgery. Previous work investigating a targeted histotripsy technique in a porcine model of deep vein thrombosis found blood flow improvements with limited hemolysis, spared vessel walls and venous valves, and no signs of pulmonary embolism, pointing toward the potential of histotripsy as a safer alternative to conventional techniques.^31^ Our system's precise focus minimized scatter of ultrasound energy outside the focal zone, which may allow for the preservation of surrounding tissue.

Existing ultrasound-based thrombolysis devices like SonoLyse (Boston Scientific, Malborough, Massachusetts) and EKOS (Boston Scientific) utilize low-intensity ultrasound and thrombolytic drugs to enhance clot dissolution. SonoLyse employs ultrasound waves to agitate and disperse clot fragments, aiding the distribution of thrombolytic agents within the clot.^32^ EKOS integrates ultrasound with catheter-directed thrombolysis to deliver effectively drugs into the thrombus, which significantly improves pulmonary embolism patient outcomes.^33^ The EKOS system has been evaluated in clinical trials, including the interventional management of stroke trials, where it was shown to enhance the efficacy of thrombolytic therapy.^33^ The EkoSonic system is Food and Drug Administration (FDA)-approved and has been widely tested in clinical settings, demonstrating improvements in pulmonary artery pressure and right ventricular function after ultrasound-accelerated catheter-directed thrombolysis.^34^

Unlike other ultrasound-based treatments, histotripsy employs HIFU to mechanically disrupt clots without the need for adjunctive drug therapy. This approach targets the clot directly through cavitation, offering a drug-free alternative that could reduce the risk of bleeding and eliminate the need for systemic thrombolytics, making it a viable option for patients with contraindications to traditional drug therapies.^35^ Studies have demonstrated that histotripsy can break down large clots into smaller particles, facilitating rapid clot reduction and natural clearance from the body.^36^

Several important limitations of this study must be considered. First, it was conducted in a controlled in vitro setting, which does not account for the complexities of human anatomy, such as clot composition, bone interference, and surrounding tissue complexity. Patient-specific anatomy such as skull thickness, density, and vascular geometry can affect transcranial histotripsy efficacy, as cavitation thresholds vary with skull density ratio and treatment depths range from 5 to >16 mm depending on bone characteristics.^37^ These challenges highlight the need for personalized treatment planning using imaging and tailored ultrasound parameters. Phase aberration and attenuation effects may negatively impact the efficacy of the current set-up. Despite these limitations, our optimized ultrasound parameters provide a strong foundation for future work, including the use of multiple transducers and imaging-guided targeting.

### Future directions

To advance histotripsy toward clinical application, further exploration is needed in several areas, including in vivo testing in animal models, enhancing ultrasound focus to penetrate dense tissues such as bone, improving gel pad durability for consistent outcomes, and investigating its use for clot dissolution in ischemic stroke as a non-invasive alternative to thrombectomy or drug therapies.

The clinical translation of histotripsy faces regulatory and safety challenges, requiring rigorous studies to ensure effective clot dissolution while minimizing damage to surrounding tissues. Targeted ultrasound energy delivery and overcoming barriers such as bone and gel pad degradation are critical for safe application, making thorough testing and optimization essential for clinical integration.

## Conclusions

This study demonstrates the potential of ultrasound as a tool for clot dissolution. With optimized parameters including pulses per burst, repetition rates, and output amplitudes, clots can be effectively destroyed in artificial environments, providing a promising foundation for non-invasive stroke treatment.

## Supplemental Material

sj-docx-1-ine-10.1177_15910199251347838 - Supplemental material for Histotripsy: A novel non-invasive ultrasound technology for precision thrombolysis in acute ischemic stroke management Supplemental material, sj-docx-1-ine-10.1177_15910199251347838 for Histotripsy: A novel non-invasive ultrasound technology for precision thrombolysis in acute ischemic stroke management by Ameer E Hassan, Thomas Dreyer and Yousef Khalili in Interventional Neuroradiology
