# Supplementary material for: Histotripsy: A novel non-invasive ultrasound technology for precision thrombolysis in acute ischemic stroke management
Source: Interv Neuroradiol. 2025 Jun 10;32(5):2818–26. doi: 10.1177/15910199251347838 (PMC12151993; doi:10.1177/15910199251347838)
Supplement: Supplementary file 1 [file sj-docx-1-ine-10.1177_15910199251347838.docx]

**SUPPLEMENTAL MATERIALS**

Support structure

3-axis translational stage

Probe holder

Water tank

Transducer in support frame

**Figure S1:** Representative image of the ultrasound test setup with fixed transducer positional and moveable probe holder.

**Figure S2:** Glass model setup with auxiliary equipment to position the clot in vessel model.

**Figure S3:** Example images to assess clot size reduction rate, lower image pre-, upper post-treatment, overlay of original vs. contrast-enhanced picture (green and orange pixels, vertically shifted).
